# Peculiarities of Electric and Dielectric Behavior of Ni- or Fe-Doped ZnO Thin Films Deposited by Atomic Layer Deposition

**DOI:** 10.3390/ma17143546

**Published:** 2024-07-18

**Authors:** Albena Paskaleva, Dencho Spassov, Blagoy Blagoev, Penka Terziyska

**Affiliations:** Institute of Solid State Physics, Bulgarian Academy of Sciences, Tzarigradsko Chaussee 72, 1784 Sofia, Bulgariablago@issp.bas.bg (B.B.);

**Keywords:** doped ZnO, atomic layer deposition, electric and dielectric properties, polarization mechanisms

## Abstract

The physical properties of ZnO can be tuned efficiently and controllably by doping with the proper element. Doping of ZnO thin films with 3D transition metals that have unpaired electron spins (e.g., Fe, Co, Ni, etc.) is of particular interest as it may enable magnetic phenomena in the layers. Atomic layer deposition (ALD) is the most advanced technique, which ensures high accuracy throughout the deposition process, producing uniform films with controllable composition and thickness, forming smooth and sharp interfaces. In this work, ALD was used to prepare Ni- or Fe-doped ZnO thin films. The dielectric and electrical properties of the films were studied by measuring the standard current–voltage (I–V), capacitance–voltage (C–V), and capacitance–frequency (C–f) characteristics at different temperatures. Spectral ellipsometry was used to assess the optical bandgap of the layers. We established that the dopant strongly affects the electric and dielectric behavior of the layers. The results provide evidence that different polarization mechanisms dominate the dielectric response of Ni- and Fe-doped films.

## 1. Introduction

Zinc oxide (ZnO) is wide band-gap n-type semiconductor, which has been intensively investigated over the years because of its favorable chemical, optical, mechanical, and electrical properties, enabling its application in a broad range of opto-electronic, micro-electronic, nano-electronic, and acousto-electronic applications [1]. ZnO can be obtained with a wide variety of morphologies, which constitute the wide diversity of its properties. The structure and morphology of ZnO layers, as well as their properties, are strongly dependent on the deposition technique and conditions. It should be noted that ZnO can be obtained using almost all deposition methods, e.g., chemical vapor deposition, sputtering, atomic layer deposition, pulsed laser deposition, sol-gel spin coating, spray pyrolysis, etc. [2,3,4,5,6,7]. In addition, efficient control over its morphology, structural, optical, electrical, and magnetic properties can be established through doping with different elements [8,9,10,11,12]. Atomic layer deposition (ALD) is the most advanced technique, which enables high accuracy in the deposition process, excellent film uniformity, control over the thickness (down to several nm), and smooth and sharp interfaces. In ALD, monolayer growth is achieved via a self-limiting surface reaction between precursors, which are sequentially pulsed in the chamber. This technique is also intensively used to deposit thin ZnO films [13,14,15,16,17], including their doped counterparts [18,19,20]. In other words, by using the proper deposition method and deposition conditions and by doping with the proper element, it is possible to realize ZnO films with flexible and versatile properties, satisfying particular requirements and enabling a wide range of possible devices and applications, such as optical waveguides, photodetectors, thin-film transistors, piezoelectric transducers, light-emitting diodes, different types of sensors, photovoltaic cells, surface acoustic wave devices, transparent conductive oxides, etc. 

From this point of view, doping with transition metal (TM) atoms, which have partially filled d-states (e.g., Fe, Co, and Ni) is of particular interest. This doping can enhance the magnetic properties of the films and facilitates the creation of diluted magnetic semiconductors, capable of exhibiting ferromagnetism at room temperature. Hence, they hold great promise for applications in spintronics [21,22,23]. However, it should be noted that despite the increased interest and research in doped ZnO films deposited by ALD, there have been relatively few investigations on transition metal-doped ZnO films [24,25]. Recently, in a number of works [26,27,28,29], we have reported results on the structural, optical, magneto-optical, dielectric, etc., properties of TM-doped ZnO layers deposited by ALD. A very strong magneto-optical Kerr effect (MOKE) has been observed in all TM-doped (Fe, Co, and Ni) ZnO films. It has been demonstrated that this effect is induced by the magnetic nature of the dopants and increases with the number of free 3D electron spins [27]. In addition, ferroelectric-like behavior has been observed in Fe-doped ZnO layers [27], providing promise for the realization of multiferroic functionalities in this material. 

In this work, atomic layer deposition (ALD) was used to prepare transition metal-doped (Ni or Fe) ZnO thin films, and their electric and dielectric properties were studied depending on the doping element. Spectral ellipsometry was used to assess the optical bandgap of the layers. We leveraged the distinctive electric and dielectric behaviours of the doped ZnO layers to draw conclusions on the dominating polarization mechanisms and to relate them to structural changes in the layers induced by doping. 

## 2. Materials and Methods

Transition metal (TM) (Ni or Fe)-doped ZnO films were prepared using a standard thermal ALD process (Beneq TFS-200 reactor system). Diethylzinc (DEZ), as a Zn precursor, and deionized (DI) water, as an oxidant, were used to deposit the ZnO matrix. The doping was performed using a metallocene (MCp_2_ = NiCp_2_ or FeCp_2_) precursor and ozone O_3_. The solid metallocene precursors were supplied by HS-300 hot source containers. The metallocene precursor was heated up to 80 °C in order to enhance the sublimation process and its vapor pressure. The deposition was performed at 180 °C and consisted of 24 ZnO/TM supercycles. Each ZnO/TM supercycle consisted of 16 cycles of DEZ/DI H_2_O, followed by 5 cycles of MCp_2_/O_3_. Pure nitrogen was used for purging after each precursor and oxidant pulse. The pulse duration and purging times (p) for both subcycles were as follows: DEZ/p/DI H_2_O/p = 0.2/2/0.2/2 s and MCp_2_/p/O_3_/p = 2/4/1/5 s. More details on the deposition of doped ZnO layers can be found in [27]. The doped ZnO films were deposited on TiN/SiO_2_/p-Si (100) substrates. The TiN layers were deposited via rf sputtering and served as the bottom electrodes. The top Pt electrodes (circles with a diameter d = 500 µm, i.e., area *A* = 1.96 × 10^−3^ cm^−2^) were deposited through a shadow mask. 

**Figure 1:**
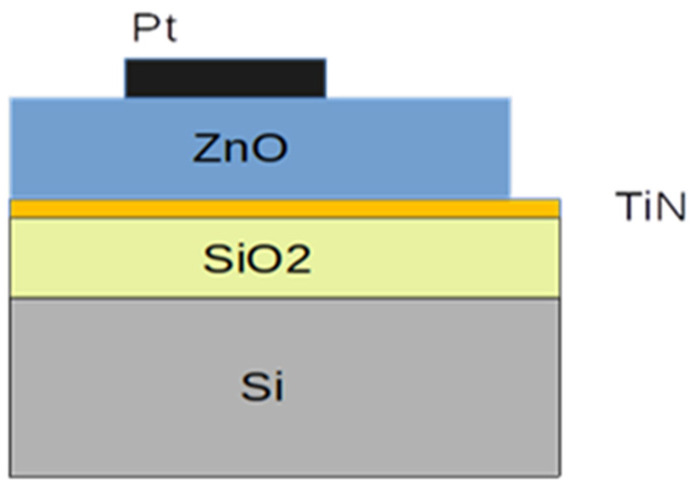
A schematic representation of the MOM structures with doped ZnO and Pt (top) and TiN (bottom) metal electrodes.

A Woollam M2000D spectroscopic ellipsometer was used to perform spectral ellipsometry investigations of the ZnO layers in the wavelength range from 193 to 1000 nm. The thickness of the layers was also measured and was about 75 nm. Electrical measurements were performed on the fabricated metal-doped ZnO metal (MOM) structures with a bottom electrode of TiN and a top electrode of Pt (Figure 1). To assess the dielectric properties of doped ZnO films, capacitance–requency (C–f) and capacitance–voltage (C–V) curves were measured (LCR meter Agilent E4980A) in frequencies ranging from 10^3^ to 10^6^ Hz at different temperatures. Temperature-dependent current–voltage (I–V) characteristics were also measured to evaluate electrical conduction in the films.

## 3. Results and Discussion

**Figure 2:**
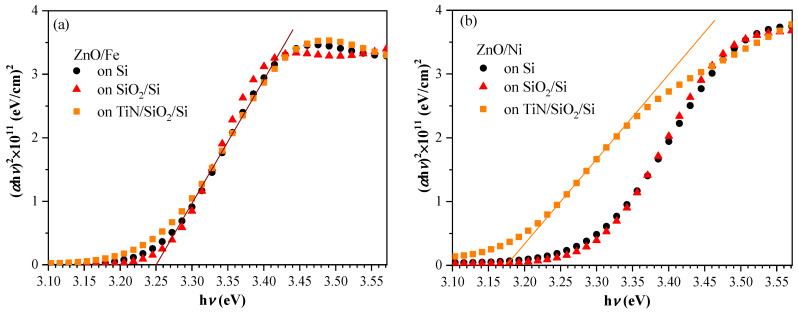
Tauc plots of (**a**) Fe-doped and (**b**) Ni-doped ZnO layers deposited on various substrates.

Spectral ellipsometry measurements were used to determine the optical bandgap *E_g_* of Ni- and Fe-doped ZnO layers deposited on three different substrates (Si, Si/SiO_2_, and Si/SiO_2_/TiN) using a Tauc plot (Figure 2). Bearing in mind that ZnO is a direct band-gap semiconductor, the following equation, which relates *E_g_* and the absorption coefficient α, was used:

(1) α hν=A(hν−Eg)12

where *A* is a constant and *hν* is the energy of the incident light. *E_g_* was determined from the intercept of (*αhν*)^2^ with the *x*-axis. In Table 1, the obtained values of *E_g_* on the three substrates are given.

As observed, the ZnO:Ni layers deposited on Si or SiO_2_ exhibited a slightly larger bandgap of −3.30 eV compared to ZnO/Fe layers, with a bandgap estimated around 3.26–3.27 eV. A similar bandgap was obtained for ZnO when deposited on TiN. However, the *E_g_* of ZnO/Ni decreased significantly for the layers deposited on TiN, implying an increase in defect-induced near-band-edge transitions. This indicates that the deposition of ZnO/Ni is affected by the TiN surface and proceeds in a different manner compared to Si or SiO_2_ surfaces. It is very likely that the composition and/or microstructure of the ZnO/Ni films close to the interface with TiN exhibit some variations compared to the bulk of the film. For pure ZnO deposited on Si, the *E_g_* value determined using ellipsometry is 3.33 eV [26], i.e., for both Ni- and Fe-doped films, the obtained *E_g_* is smaller than that of pure ZnO. The shrinkage in the optical bandgap of the doped ZnO layers is related to strong exchange interactions between the s and p electrons of the host ZnO matrix and the d electrons of the dopant, indicating the incorporation of the dopant into the ZnO lattice. This conclusion is also supported by comparisons with bandgap values of about 3.29 eV obtained for similar, more lightly doped layers deposited on Si [26]. Therefore, for Fe-doping, as the doping level increases, the value of *E_g_* decreases. A similar decrease in bandgap with increasing doping has been obtained in other works and is attributed to sp–d hybridization [30,31,32]. For Ni-doped layers, the *E_g_* values are nearly the same for the two levels of doping, which is most likely due to the two competing processes: the sp–d exchange interaction, which causes a red shift, and the Burstein–Moss effect, which is due to an increase in the free carrier concentration [33] and results in a blue shift. 

**Figure 3:**
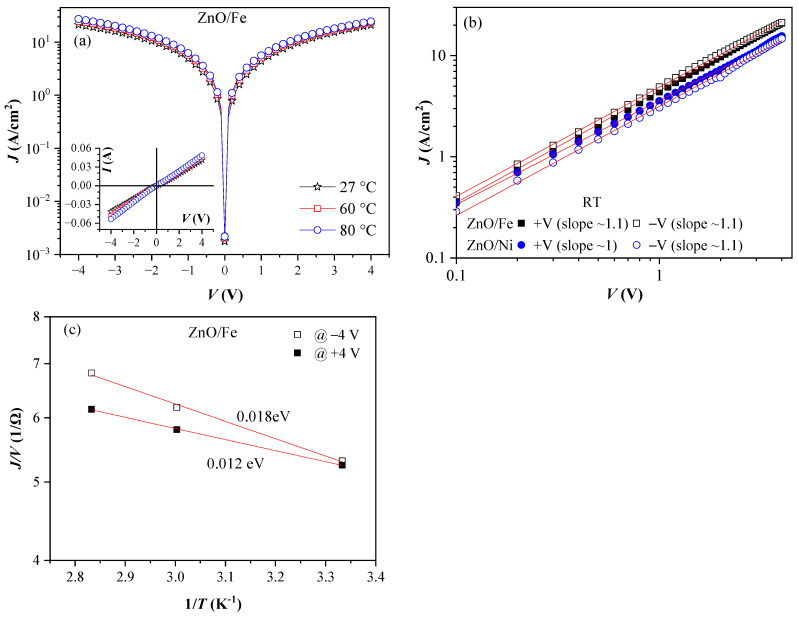
(**a**) J–V characteristics of Fe-doped ZnO layers measured at different temperatures; (**b**) J–V characteristics of doped ZnO layers at room temperature on a log-log scale; and (**c**) an Arrhenius plot for the ZnO/Fe layers.

Measurement of the I–V characteristics were performed across temperatures ranging from room temperature to 80 °C. Weak temperature dependence across the studied temperature range was observed. This was better expressed for the Fe-doped ZnO layers, where the current exhibited a slight monotonic increase with temperature (Figure 3a). The resistivity of the layers decreased with increasing temperature, indicating that the electrical transport followed typical semiconductor behavior. As is seen in the inset graphs and from the representation of the characteristics in the logI-logV coordinates (Figure 3b), demonstrating a slope close to 1, the current–voltage dependence is almost linear, i.e., J=σV, revealing dominating ohmic conduction.

The thermally activated conductivity *σ*(*T*) in semiconductors is usually assigned to the electrons hopping from the donor levels to the conduction band or from the valence band to the acceptor levels, and obeys the Arrhenius equation [34], i.e.,

(2) $$\sigma (T)=A\;exp\left(-\frac{E_{a}}{kT}\right),$$

where *E_a_* is the activation energy and can be obtained from the Arrhenius plot of I–V characteristics (i.e., ln(J/V) vs. (1/T)) (Figure 3c). For the Fe-doped ZnO layers, the determined values of *E_a_* at a voltage of ±4 V were 18 meV and 12 meV at negative and positive bias, respectively. The interpretation of *E_a_* depends on the type and concentration of impurity elements. In general, this is the difference between the bottom of the conduction band and the Fermi level. In the case of doping with one type of element and the temperature at which the condition ${\left(4N_{d}n_{i}\right)}^{1/2}\gg 1$ is satisfied (which is fulfilled here as n_i_ ≈ 10^6^ cm^−3^ for ZnO at 300 K), *E*_a_ = ϕ_d_/2, where ϕ_d_ is the depth of the impurity level measured from the bottom of the conduction band. In the case of compensation (i.e., the presence of both donor and acceptor impurities N_d_ < N_a_), E_a_ = ϕ_d_. Therefore, the obtained low activation energy indicates an impurity level located very close to the conduction band, facilitating easy ionization of this level, which explains the observed high conductivity.

**Figure 4:**
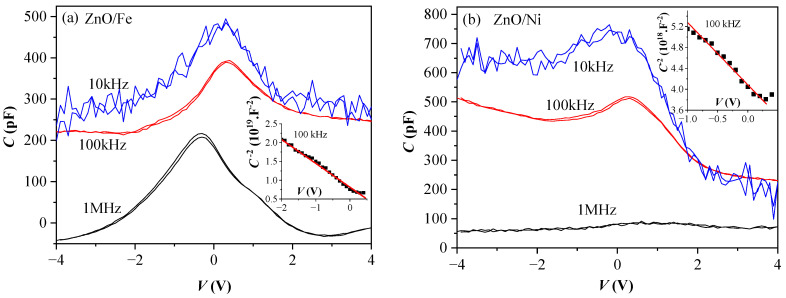
C–V characteristics of: (**a**) Fe-doped and (**b**) Ni-doped ZnO MOM structures measured at three different frequencies. In the insets, representations of the C–V curves in C^−2^ vs. V coordinates are shown.

The capacitance–voltage (C–V) characteristics of ZnO-doped capacitors were measured at three different temperatures and at different frequencies. Figure 4 shows the results obtained at room temperature for three frequencies (10 kHz, 100 kHz, and 1 MHz). It can be seen that capacitance (C) depends strongly on both the value of the applied voltage (V) and the frequency of the measurement signal. The noise in the characteristics measured at 10 kHz is related to the capture and emission processes of current carriers in defect states within the layers. At high frequencies, these processes are not detected as they cannot follow the voltage changes. In addition, the type of dopant strongly affects the C–V characteristics. Generally, the C–V characteristics for both ZnO/Fe and ZnO/Ni exhibit strong frequency dependence and a distinct peak at low positive voltage (0.3–0.5 V). As ZnO is a wide bandgap semiconductor, such behavior could be interpreted with a different type of conductivity (electronic or hole) depending on the dopant [35]. The effect of Schottky barriers at both metal electrodes is considered responsible for the presence of a peak in the C–V curves. The nearly constant values of capacitance observed for ZnO/Fe layers at voltages higher than about 2 V are associated with the full depletion-like state of the semiconductor. The increase in capacitance at negative applied voltages observed for the ZnO/Ni sample could be attributed to the presence of defects and/or the influence of parasitic contact resistance. The following equations describe the C–V characteristics of structures with a Schottky transition at the metal–semiconductor interface [35]:

(3) $$\frac{1}{C^{2}}=\frac{2}{q\varepsilon A^{2}N_{D}}(V_{bi}-V)$$

(4) $$\Phi_{b}=V_{bi}+\frac{kT}{q}ln(\frac{N_{c}}{N_{D}})$$

where *Φ_b_* is the barrier height, N_D_ is the concentration of current carriers, *A* is the area of the electrode, *V_bi_* is the built-in potential, and *N_C_* is the density of states in the conduction band at 300 K. Representation of the C–V curves in C^−2^ vs. *V* coordinates should give a straight line, which is indeed the case (see the insets in Figure 4a,b). From the obtained straight lines, the following values have been determined: for Fe-doped ZnO, N_D_ = 6.83 × 10^17^ cm^−3^, *V_bi_* = 1.29 V, and *Φ_b_* = 1.24 eV; and for Ni-doped ZnO, *N_D_* = 3.63 × 10^18^ cm^−3^, *V_bi_* = 3.43 V, and *Φ_b_* = 3.4 eV. As is seen, a higher dopant concentration was obtained for the ZnO/Ni layers, which is in agreement with our previous studies [26], indicating that at equal deposition conditions, higher concentrations of Ni are incorporated in ZnO films compared to Fe. Generally, the barrier height can be calculated as the difference between the work function of the metal electrode (Pt) and the electron affinity of ZnO. The electron affinity of ZnO is about 4.2 eV and the work function of Pt is 5.65 eV. Hence, the barrier height should be Φ_b_ = 5.65 eV − 4.2 eV = 1.45 eV [36]. Therefore, the obtained barrier height for the ZnO/Fe layers agrees with this value, which proves the formation of Schottky contact at the Pt/ZnO/Fe interface. An unrealistically high value for Φ_b_ was obtained for ZnO/Ni films, which can be attributed to various barrier inhomogeneities and/or surface defects. 

**Figure 5:**
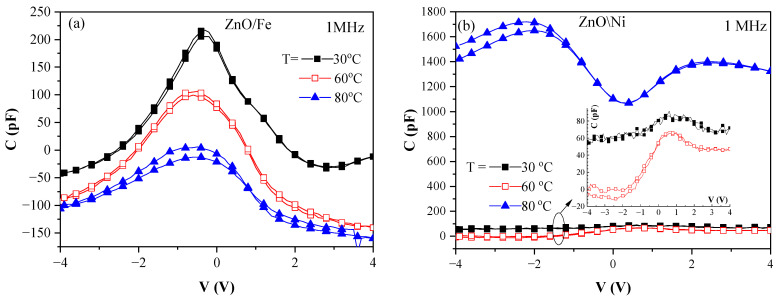
C–V (1 MHz) characteristics of MOM capacitors with (**a**) Fe-doped and (**b**) Ni-doped ZnO layers measured at various temperatures.

Strong dependence on the dopant is unambiguously revealed by the temperature dependence of the C–V curves measured at 1 MHz. With increasing temperature, the shape of the C–V curve in the case of Fe doping was preserved, and for the layers doped with Ni, the shape changed significantly (Figure 5). Moreover, in the case of ZnO/Fe the capacitance decreased with increasing temperature, whereas for ZnO/Ni it decreased at 60 °C, but at 80 °C, a strong increase (about one order of magnitude) was observed. It should be noted (Figure 4 and Figure 5) that negative capacitance values were observed under certain measurement conditions (e.g., for Fe-doped ZnO at 30 °C for voltages |V| > 2 V, whereas at 80 °C, negative capacitance was observed in the whole voltage range; and for ZnO:Ni, this region was observed only for V < −1 V at T = 60 °C). This will be commented on later.

**Figure 6:**
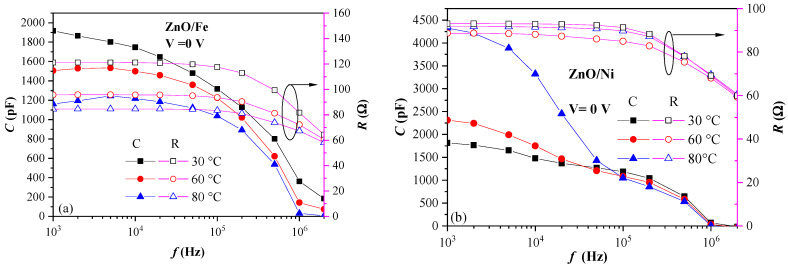
Capacitance dispersion of metal/ZnO/metal structures at different temperatures for (**a**) Fe-doped ZnO and (**b**) Ni-doped ZnO. The change in resistance with frequency is also presented (hollow symbols, right-hand y-axis).

**Figure 7:**
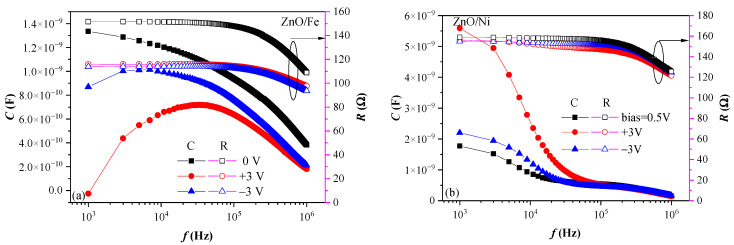
Capacitance dispersion of metal/ZnO/metal structures at different applied voltages for (**a**) Fe-doped ZnO and (**b**) Ni-doped ZnO. The change in resistance with frequency is also presented (hollow symbols, right-hand y-axis).

The dielectric properties of the layers were examined by measuring the change in capacitance with frequency (C–f curves). The increase in capacitance (dielectric constant) with decreasing frequency, *f*, is referred to as “dielectric relaxation” and is attributed to the operation of various polarization mechanisms dependent on *f*. Several polarization mechanisms (ionic polarization, dipole/orientation polarization, and space charge polarization) give rise to increased capacitance when frequency is decreased [37]. The results reveal the existence of very complex dielectric phenomena in the ZnO layers, which are strongly affected by the dopant, as well as by the measurement conditions (e.g., frequency, temperature, and applied voltage). To obtain more in-depth insights into these phenomena, the dispersion of dielectric properties were measured in a frequency range of 1 kHz–1 MHz at three temperatures (Figure 6) and at different applied voltages (0, +3, and −3 V) (Figure 7). It should be noted that according to simulations performed in [38], the edge effects that may arise from asymmetry of the electrodes should not result in significant error in the measured capacitance of structures with a thickness and an area similar to ours (e.g., t = 75 nm and d = 500 µm).

The dielectric response of the studied doped ZnO layers depends very strongly on the doping element. First, the dielectric behavior of the curves measured at different temperatures and V = 0 V were considered (Figure 6). Very strong dependence was observed on the doping element. Furthermore, the capacitance decreased with temperature for Fe-doped ZnO (Figure 6a) and increased with temperature for the Ni-doped layers (Figure 6b).

For ZnO/Fe (Figure 6a), with increasing frequency in the range of 10^3^–10^5^ Hz, capacitance only slightly changed. Dipolar polarization is usually considered the dominant polarization mechanism in this frequency range [37]. At high frequencies (*f* > 10^5^ Hz), the dipole rotation and their alignment with the applied AC field does not follow the field changes, leading to the observed rapid decrease in capacitance. As discussed above, the E_g_ of the ZnO/Fe layers decreased with increasing doping levels, which signifies the incorporation of Fe into the ZnO lattice. Therefore, we suggest that Fe-O bonds are formed when Fe is substituted at Zn sites and the rotation of these bonds is at the origin of the enhanced dielectric response of the layer [39]. The domination of orientation polarization at 10^3^–10^5^ Hz was confirmed via the temperature dependence of capacitance, which decreased with increasing temperature (Figure 6a). This is easily explained as the increase in the thermal energy of the dipoles counteracts their alignment with the applied field. The Ni-doped layers demonstrated substantially different dependence in their dielectric behavior on frequency and temperature. Unlike the ZnO/Fe layers, ZnO/Ni reveals increased capacitance in the frequency range of 10^3^–10^4^ Hz, as compared to capacitance at *f* = 10^4^–10^5^ Hz (Figure 6b). This is a clear indication that an additional polarization mechanism exists at lower frequencies and is associated with polarization at nanocrystalline grain boundaries and/or heterogeneous interfaces. This mechanism is also known as Maxwell–Wagner (MW) polarization and occurs at the interfaces between two media with distinctly different conductivity, e.g., highly conducting grains and relatively lower conducting grain boundaries or heterointerfaces. The difference in conductivity results in an accumulation of electrons at the boundaries between the two media, producing a charge buildup at the internal interfaces. Consequently, an increase in capacitance was observed. The increase in capacitance of the Ni-doped layers in the frequency range of f = 10^3^–10^4^ Hz was observed at all temperatures and confirmed the presence of an additional polarization mechanism in these layers, as mentioned above. Moreover, strong temperature dependence of the C–f curves of the ZnO/Ni layers at *f* = 10^3^–10^4^ Hz was observed and this was opposite to that of the ZnO/Fe films, i.e., capacitance increases with increasing temperature, which further supports the suggestion of the dominance of MW polarization in the ZnO/Ni layers. At *f* = 10^4^–10^5^ Hz, the dependence on temperature becomes weaker, and at frequencies around 10^5^ Hz, it reverses, i.e., capacitance slightly decreases as temperature increases, indicating a shift in the dominating polarization mechanism from MW to dipole polarization. MW polarization, due to charge accumulation at grain boundaries, is well justified, bearing in mind that our previous studies revealed that the doped ZnO films have polycrystalline hexagonal wurtzite structures [26,27]. However, the two doped films have similar grain sizes (23 nm for Fe-doped and 28 nm for Ni-doped ZnO), whereas the increase in capacitance at frequencies of *f* = 10^3^–10^4^ Hz was observed only for the later. Therefore, an additional reason for increased MW polarization in the Ni-doped layers should exist. 

The dependence of dielectric relaxation on the applied voltage (Figure 7) was examined to obtain greater insight into the influence of both metal/ZnO interfaces. It is seen that the two types of doping had distinctly different effects on the C–f curves measured at positive or negative applied voltages. In the case of Ni doping, the capacitance increased when the voltage (irrespective of polarity) was applied (Figure 7b), whereas for Fe-doped ZnO, the capacitance decreased (Figure 7a). Generally, the shape of the C–f curves did not change (except for Fe-doped ZnO at low frequencies) and was similar to the respective curves measured at 0 V, which implies that the dominating polarization mechanisms do not change upon the application of voltage. For both types of doping, the change in capacitance was always stronger for positive applied voltage, especially at lower frequencies. At positive voltage, the electrons are injected from the bottom TiN electrode. Therefore, the electrical quality of the bottom electrode interface with ZnO, e.g., the existence of inhomogeneities, defects, traps, etc., substantially affects dielectric relaxation. For Ni-doped ZnO layers, this result is in very good agreement with the observed decrease in bandgap when deposited on TiN. This gives us a reason to conclude that the MW space charge relaxation in ZnO/Ni layers is mostly due to structural changes/inhomogeneities and/or defects near the ZnO/TiN interface. This may arise from the formation of NiO clusters in the ZnO matrix. In our previous study on more lightly Ni-doped ZnO, the formation of NiO clusters was observed [26]. Deposition on TiN could result in increased formation of NiO clusters close to the TiN interface, which affects both the bandgap and the polarization processes.

Finally, the observed negative capacitance (i.e., negative permittivity) in some cases, as revealed in Figure 5, Figure 6 and Figure 7, should be addressed. Negative permittivity in different materials measured at radio frequencies is often reported, including cases where Ni or Fe are incorporated into a particular host matrix [40,41]. Usually, the appearance of negative permittivity at high frequencies is explained within the Drude model, which considers the combined contributions of delocalized free electrons, as well as charges localized by oxygen vacancies and other defects [42]. Negative capacitance has also been reported in ZnO-based materials and Schottky structures [43], and is usually observed at low frequencies for forward biased structures. The loss of interface charge states, together with the presence of oxygen vacancies, as well as piezoelectric and electrostriction effects, are believed to be the basis of the observed negative capacitance phenomena [44]. The negative capacitance values are also linked to processes initiated by injected hot electrons at the metal-semiconductor junction [43]. The reasons for the appearance of negative capacitance regions in the C–V and C–f curves of our Ni- and Fe-doped ZnO structures are not clear at the moment and require more dedicated measurements and analysis to clarify their origin and dependence on the doping. This is beyond of the scope of this study. Nevertheless, the more pronounced negative capacitance behavior of Fe-doped ZnO suggests that it might be related to their stronger ferromagnetic behavior, as revealed by MOKE [27]. 

## 4. Conclusions

Ni- or Fe-doped ZnO layers were prepared using ALD, and their electric and dielectric properties were studied depending on the doping element. The results reveal the complex nature of the polarization processes that take place in the layers, depending on their composition and the measurement parameters (temperature, frequency, and applied voltage polarity). In Fe-doped ZnO, dipolar polarization is the dominant polarization mechanism, whereas Maxwell–Wagner space-charge polarization contributes substantially to dielectric polarization in ZnO/Ni films. The decrease in optical bandgap with increasing Fe doping indicates effective incorporation of Fe into the ZnO lattice. Ni-doped ZnO films demonstrate some peculiarities compared to Fe-doped films, e.g., decreased bandgap when deposited on TiN, unrealistically high barrier heights at the Pt/ZnO/Ni interface, and increased interfacial MW polarization. These are attributed to the existence of defects and inhomogeneities close to the metal/ZnO interfaces, likely involving the formation of NiO clusters within the layers.

The obtained results provide insights into the electrical and dielectric behavior of doped ZnO layers and, in combination with our previous studies, reveal the very promising properties of Fe-doped ZnO layers. In particular, the evidence for better incorporation of Fe into the ZnO matrix and the dominant dipolar polarization explains the stronger ferromagnetic response and ferroelectric-like behavior of Fe-doped films compared to Ni-doped films. For Ni-doped films, the ALD scheme should be optimized to avoid inhomogeneities and interfacial defects. In addition, more precise measurements and analyses are required to clarify the origin of the observed negative capacitance in some cases and whether it is a real phenomenon or a measurement artefact. This could expand the diverse application areas of these layers. 

## Figures and Tables

**Table 1:** The band-gap values of Ni- and Fe-doped ZnO layers deposited on different substrates.

	E_g_, eV on Si	E_g_, eV on SiO_2_/Si	E_g_, eV on TiN/SiO_2_/Si
ZnO/Ni	3.30	3.30	3.17
ZnO/Fe	3.26	3.27	3.25

## Data Availability

The datasets that support the findings in this study are available from the corresponding author upon reasonable request.
